# A Systematic Review and Meta-Analysis of Buyang Huanwu Decoction in Animal Model of Focal Cerebral Ischemia

**DOI:** 10.1155/2013/138484

**Published:** 2013-06-04

**Authors:** Rui-li Wei, Hai-juan Teng, Bo Yin, Yang Xu, Yue Du, Fang-pin He, Ke-tan Chu, Ben-yan Luo, Guo-qing Zheng

**Affiliations:** ^1^Brain Medical Center, First Affiliated Hospital of Zhejiang University, School of Medicine, Hangzhou Zhejiang 31000, China; ^2^The Center of Neurology and Rehabilitation, The Second Affiliated Hospital of Wenzhou Medical College, Wenzhou 325027, China

## Abstract

Buyang Huanwu Decoction (BHD) is a well-known Chinese herbal prescription for ischemic stroke. The objective of this systematic review and meta-analysis is to provide the current evidence for neuroprotective effects of BHD and its possible mechanisms in animal models of focal ischemia. A systematic literature search, through October 2012, was performed using six databases. The outcome measures assessed were infarct size and/or neurological score. Fifty-six studies with 1270 animals that met the inclusion criteria were identified. The median score for methodological quality was 3 with a range of 2 to 6. Compared with vehicle or no treatment controls, BHD gave a 37% improvement in outcome for all doses ranging from 1.0 g/kg to 60 g/kg at each time point that BHD was administered (*P* < 0.01). Efficacy was higher in mouse models that utilized suture occlusion and temporary ischemia. The neuroprotective effects of BHD are involved in multiple mechanisms and act upon multiple cell types. In conclusion, BHD possesses substantial neuroprotective effects in experimental stroke probably as a result of the multitarget therapy strategy typically utilized in traditional Chinese medicine. Future research should examine the presence of possible experimental bias and an in-depth study of herbal compound preparations.

## 1. Introduction

More than 1,000 drugs have been tested in experimental stroke, with nearly half reporting efficacy in animal models of focal cerebral ischemia [1]. However, only tissue plasminogen activator has shown proven efficacy in human studies. Presently, no single drug is considered to be universally neuroprotective in cerebral ischemia, and current guidelines for poststroke treatment remain controversial [1, 2]. It is therefore necessary to investigate alternative and frequently overlooked potential treatments, of which traditional Chinese medicine (TCM) occupies a substantial proportion.

Buyang Huanwu Decoction (BHD) is a well-known and classical TCM herbal prescription for ischemic stroke and has been used during poststroke rehabilitation for more than 300 years [3]. BHD is composed of seven kinds of Chinese herbs: Huangqi (Radix Astragali seu Hedysari), Danggui (Radix *Angelica sinensis*), Chi Shao (Radix Paeoniae Rubra), Chuanxiong (Rhizoma Ligustici Chuanxiong), Honghua (Flos Carthami), Taoren (Semen Persicae), and *Dilong* (*Pheretima*). All of the aforementioned compounds are recorded in Chinese Pharmacopoeia. From a TCM perspective, BHD use during ischemia invigorates the body, enhances blood circulation, and activates Qi flow through energy meridians [3, 4].

Recent studies have reported that BHD has neuroprotective effects and is effective against cerebral ischemia-reperfusion (CI/R) injury in humans and animal models [5–12]. Mechanistically, BHD has been shown to exert its neuroprotective effect by promotion of growth and differentiation of neural cells [5, 13], inhibition of apoptosis [5], repression of inflammatory reactions [5], and reduction of Ca^2+^ overload [14] and oxidative stress/nitration stress reaction [15, 16]. The broad range of action attributed to BHD suggests that the neuroprotective effects of BHD on brain ischemia are through multiple mechanisms. However, demonstration of efficacy and mechanisms of neuroprotection of BHD still lack systematic analysis in experimental stroke, and the current clinical evidence is insufficient to support a routine use of BHD for acute ischemic stroke due to the poor methodological quality [12]. 

In this paper, we report a systematic review and meta-analysis of the use of BHD in animal models of experimental stroke. The objectives of the present study were to

1. systematically review and collate the experimental evidence for BHD administered before or after onset of focal cerebral ischemia in animal models;
2. determine the efficacy of BHD in focal cerebral ischemia and explore the impact on that efficacy of defined *in vivo *characteristics;
3. systematically analyze the possible neuroprotective mechanisms of BHD;
4. propose the development of further preclinical hypotheses to test in animals and ultimately aid in the design of future large-scale clinical trials in human patients.

## 2. Materials and Methods

### 2.1. Database and Literature Search Strategies

Studies of BHD in animal models of stroke were identified from PubMed, Embase, *Biosis*, China National Knowledge Infrastructure, VIP Database for Chinese Technical Periodicals, and Chinese Biomedical Literature Database. All of the searches were performed through October 2012. Our search strategy included the following words and phrases: “Buyang Huanwu” OR “Bu yang Huan wu” OR “Bu-yang Huan-wu” AND “isch(a)emia” OR “stroke” OR “infarct” OR “middle carotid artery occlusion (MCAO).” All searches were limited to studies on animals. Reference lists from the resulting publications and reviews were used to identify further relevant publications. 

### 2.2. Inclusion Criteria

To prevent bias, inclusion criteria were prespecified as the following: (1) experimental ischemic stroke was induced in rodents by transient ischemia (temporary MCAO or embolic stroke) and permanent MCAO. (2) BHD was administered as originally described in “Yi Lin Gai Cuo” (Correction of Errors in Medical Classics). BHD was composed of *Astragalus membranaceus* (extracted from *Astragalus* root), *angelica archangelica* (from Chinese *Angelica* root), *Paeonia lactiflora* (Red Peony root), Rhizoma Ligustici Chuanxiong (Szechuan Lovage root), Semen persicum (peach seed); Gencos (safflower), and *Lumbricus* (earthworm). (3) There was no administration of any other agent with potentially neuroprotective effects. (4) Infarct size or neurobehavioral scores were compared with those of control animals receiving vehicle or no treatment. (5) A control group was included in the study design.

Pre-specified exclusion criteria were models of nonfocal cerebral ischemia (traumatic models, global models, and hypoxic-ischemic models), a modified formula of BHD, no control group, or duplicate publications.

### 2.3. Data Extraction

**Figure 1:**
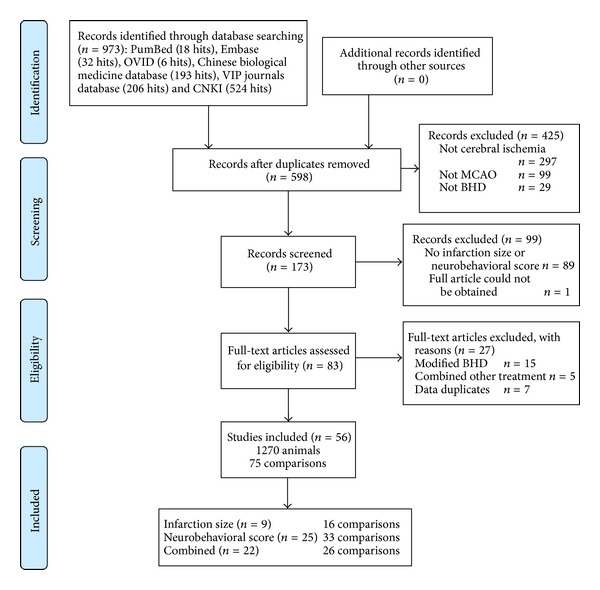
Flowchart of study selection process.

Two authors (Rui-li Wei and Hai-juan Teng) extracted data from the included trials independently, based on the inclusion criteria (Figure 1). According to the methodology described by Macleod et al. [17, 18], a “comparison” is defined as the assessment of outcome in treatment and control groups after treatment with an administered dose of drug or vehicle, with treatment commencing at a given time before or after the induction of cerebral ischemia. For each comparison, data was collected for mean outcome, standard deviation (SD) and the number of animals per group. In the event of missing data concerning the meta-analysis, authors of the original paper were contacted for additional information. If data were only expressed graphically, the numerical values were requested from the authors, and if a response was not received, digital ruler software was utilized to measure graphical data. If data required for meta-analysis were lacking, the studies were excluded from the analysis altogether. In instances of multiple-dose BHD administration, comparisons were grouped according to the first dose at initial administration with the administered dosage recorded as the total dose in the first 24 hours after ischemia. If neurological tests were performed at different times, only the final test was included. If one group of animals was assessed in more than one neurological domain (e.g., motor and sensory scores) or both neurological score and infarct size were measured, data were combined using meta-analysis (below) for an overall estimate of effect magnitude and standard error. Effect size is defined as the proportional improvement in outcomes (infarct size, neurologic score, or combined score) in treated animals relative to untreated ischemic controls.

### 2.4. Quality of Evidence

Study quality was assessed using the following criteria [18, 19]: (1) publication after peer review, (2) statement of temperature control, (3) random allocation to treatment or control groups, (4) masked induction of ischemia, (5) masked assessment of outcome, (6) use of anesthetic without significant intrinsic neuroprotective activity, (7) appropriate animal model (aged, diabetic or hypertensive), (8) sample size calculation, (9) compliance with animal welfare regulations, and (10) statement of potential conflict of interests. Each study was given a quality score out of a maximum total of ten points, and the group median was calculated.

### 2.5. Statistical Analysis

Data were processed as described previously [18]. Briefly, for each comparison, the mean outcome for the treatment group and the standard deviations in treatment and control groups were expressed as a proportion of the outcome in the control group, and the effect size (the difference between the treatment and control groups) and its standard error were calculated. Data were aggregated using a weighted mean difference with the random effects model of inverse variance method, a more conservative technique than fixed-effects meta-analysis. To explore the impact of study characteristics on estimates of effect size, we then performed a stratified meta-analysis with experiments grouped according to the following: study quality, time of administration, drug dose, route of drug delivery, duration of ischemia, method of induction of ischemia, time to outcome measurement, outcome measurement methods, whether the data had been published or unpublished, and species and gender of animal used. The presence of publication bias was visually assessed by producing a funnel plot and asymmetry test by using Stata Software (Stata version 11.0).

Significant difference between groups was assessed by partitioning heterogeneity and using the *χ* ^2^ distribution with *n* − 1 degrees of freedom, where *n* equals the number of groups. To allow for multiple comparisons, we set our significance level at *P* < 0.001.

## 3. Results

### 3.1. Study Inclusion and Characteristics

Based on our search criteria, we identified 173 studies that investigated the use of BHD in an animal model of focal cerebral ischemia (Figure 1). The earliest study was published in 1992 [67]. Out of the 173 studies identified from the literature searches, the final analysis included 56 studies, with 1270 experimental subjects used in total (Table 1). Male rat models (Wistar, Sprague-Dawley) were used in 49 of the 56 studies, and mixed gender Wistar and Sprague-Dawley (SD) rats were used in two studies, while the remaining three studies used a male Kunming (KM) mouse model, ICR mouse model, and gerbil model. Forty-nine of these studies utilized the suture-occluded MCAO animal model. In the remaining studies, five studies utilized the unilateral middle cerebral artery cauterization model while the remaining two studies used photothrombotic ischemia and autologous blood clot embolic ischemia animal models. Infarct size was reported in 31 studies and neurobehavioral outcome was reported in 47 studies. Only one study analyzed the survival rates of animals after ischemia and none of the studies addressed drug side effects [5]. Within the 56 studies included here, 75 comparisons were identified. Timing of initiation of treatment ranged from seven days before to 20 days after the induction of ischemia. Effect size was measured at a median of 7 days (24 hours to 56 days) after the onset of ischemia. Ten of the studies are published in the form of an academic dissertation (not published formally).

### 3.2. Study Quality

**Figure 2:**
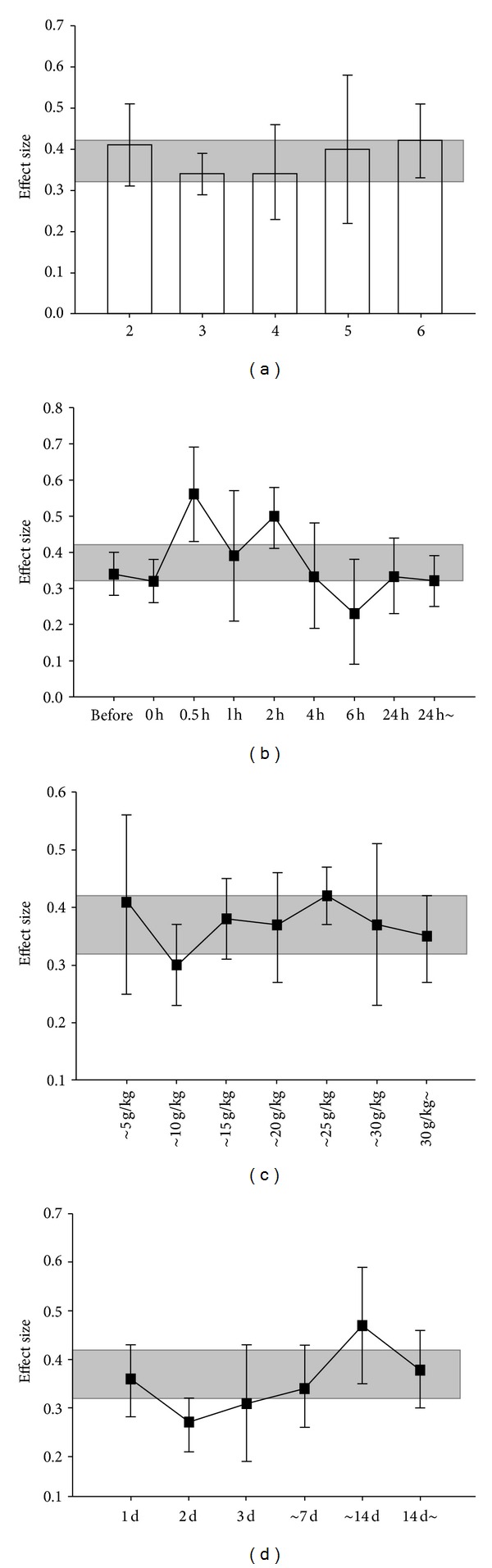
Point estimates and 95% CIs of effect size by (a) reported study quality score, (b) timing of treatment, (c) BHD dose and (d) time to outcome measurement. The 95% CI for the global estimate is shown as a grey band.

None of the studies utilized here described a sample size calculation, masked induction of ischemia, or contained a statement of potential conflict of interests. Random allocation to a treatment group was described in 51 out of the 56 studies used. Masked assessment of outcome was used in ten studies and an appropriate animal model that is relevant to the clinical situation such as aged animals, hyperglycemia, or hypertension was used only in three studies [9, 31, 32]. The median reported quality score (see Section 2) was 3 (range from 2 to 6), and classifying studies by quality score found no significant differences between high and low quality studies (Figure 2(a); *χ* ^2^  =  2.89, df = 4, and *P* = 0.58).

**Figure 3:**
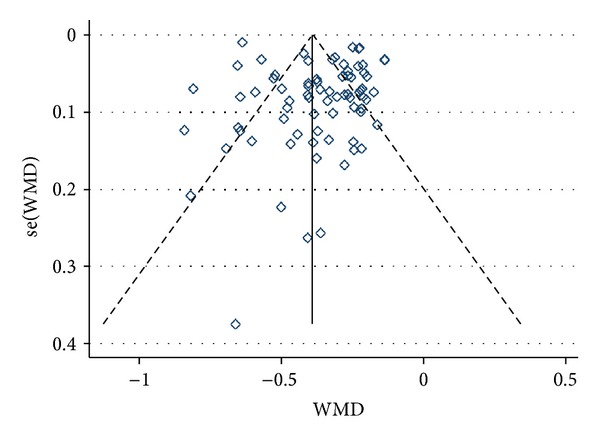
Funnel plot of the effect size of BHD treatment for animal models of focal ischemia.

The funnel plot revealed an asymmetrical distribution of studies around the line of identity, indicating the possibility of indistinctive small study bias (Egger's test, bias = 1.067193 (95%  CI = −0.3760313 to 2.510418), *P* = 0.145) (Figure 3).

### 3.3. Overall Efficacy and Impact of Time of Administration and Drug Dose

The global estimate of the effect of BHD was 0.37 (95% confidence interval (CI) 0.32–0.42, *P* < 0.00001). There was significant statistical heterogeneity (*χ* ^2^  =  1268.19,  df = 74, and *P* < 0.00001) between comparisons. Study characteristics are shown in Table 1.

Timing of initiation of treatment ranged from seven days before to 20 days after the induction of ischemia. Significant protection was seen for all time points examined. Neuroprotection was maximal when BHD was administered up to 30 minutes after MCAO (Figure 2(b); *χ* ^2^  =  27.05, df = 8, and *P* = 0.0007). Significant neuroprotective effects were noted for all doses of BHD (ranging from 1.0 g/Kg to 60 g/Kg), with a maximum effect near a dose of 25 g/Kg. However, no significant differences in dosage were determined (Figure 2(c); *χ* ^2^  =  9.54, df = 6, and *P* = 0.15). Furthermore, there was a trend for effect magnitude to be greater with the longer interval between ischemia and measurement of outcome (Figure 2(d); *χ* ^2^  =  13.29, df = 5, and *P* = 0.02); however this trend did not reach our pre-specified significance threshold.

### 3.4. Impact of the Original Study Design

**Figure 4:**
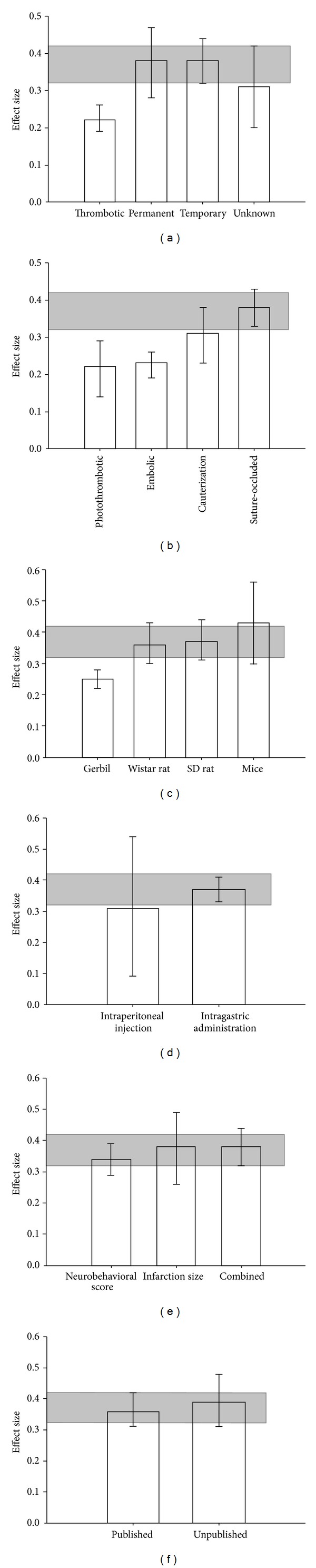
Point estimates of effect size and 95% CIs by (a) duration of occlusion, (b) method of ischemia induction, (c) route of drug delivery, (d) animal species, (e) measurement method of outcome, and (f) data published or unpublished. The 95% CI for the global estimate is shown as a grey band.

All included studies employed either a temporary or permanent ischemia model. Effect size was greater in models of temporary occlusion than in either permanent or thrombotic occlusion models (Figure 4(a), *χ* ^2^  =  25.45, df = 3, and *P* < 0.0001) and was greater in the suture-occluded model than in cauterization, embolic, or photothrombotic occlusion models (Figure 4(b), *χ* ^2^  =  26.78, df = 3, and *P* < 0.00001). Mouse models provided a higher estimate of effect size than those utilizing other animal models (Figure 4(c), *χ* ^2^  =  21.29, df = 3, and *P* < 0.0001).

The route of drug administration showed no difference on the effects of BHD (Figure 4(d), *χ* ^2^  =  0.24, df = 1, and *P* = 0.62). Additionally, the effect of BHD was not affected by the outcome measurement methods (Figure 4(e), *χ* ^2^  =  1.04, df = 2, and *P* = 0.59) or whether the study was published or unpublished (Figure 4(f), *χ* ^2^  =  0.33, df = 1, and *P* = 0.56). 

### 3.5. Possible Drug Protection Mechanism Analysis

Forty-four out of the 56 studies addressed the mechanism of BHD action (Table 2). A wide variety of possible neuroprotective mechanisms were proposed within these studies. During the acute phase of ischemia, the neuroprotective effect of BHD was attributed to improve cerebral circulation, blood flow, a reduction of cerebral edema, excitotoxicity, calcium overload, inflammation, oxidative stress, and nitrative stress and apoptosis. In the later phase of recovery, BHD promoted angiogenesis, neuronal regeneration, and synapse formation (Table 2). Many cell types, such as neurons, glial cells, endothelial cells, and blood cellular components, were implicated in mediating BHD's effect. The relationship of BHD with formation of new blood vessels and nerve regeneration was most studied (Table 2).

## 4. Discussion

### 4.1. Summary of Evidence

Treatment with BHD led to a substantial and highly significant 37% improvement in outcome, with improved outcome noted for all doses above 1.0 g/kg and at each time point studied. Maximum efficacy was seen within the first 30 minutes of ischemia onset, but BHD was effective even when administered before or 20 days after the onset of ischemia. This result is particularly striking, as the time window for most candidate neuroprotectants is narrow (usually within six hours) [68]. The potential of a much longer time window for BHD activity in comparison to other stroke drugs might be explained by its complex composition and the multimodal actions of TCM. 

 Classical neuroprotective studies typically focus on neurons and their neurotoxic environment or on one mechanism of action, such as anti-inflammation, free radical scavenging, or glutamate release inhibition. However the poststroke pathophysiological process is complex and involves multiple factors and cell types, such as neurons, glia, and vascular or inflammatory cells that undergo different types of cell death (e.g., the death of axons/white matter) [69, 70]. Inflammation, oxidative/nitrosative stress, and glutamate release in the pathophysiological process after cerebral ischemia all have dual functions: injury may be aggravated by their overreaction after ischemia, but the mechanism is essential either physiologically or for recovery [71–74]. Therefore, treatment methods acting upon an isolated pathway or via one mechanism are clearly inadequate for optimum neuroprotective effects [75]. Multitarget and multistage treatments present an exciting investigative direction in the field of cerebral ischemia treatment, such as the treatment of neurons, glia, and vascular cells (neurovascular unit) [69, 70, 76].

Our systematic review of BHD demonstrates that it is a multitargeted neuroprotective drug acting on the entire neurovascular unit, with therapeutic effects observed throughout many stages of pathophysiology following cerebral ischemia (Table 1). In the acute and subacute phases, BHD action on vascular endothelial cells affects regulation of cerebral circulation and blood flow [5, 34], inhibits the adhesion and infiltration of inflammatory cells [26, 31], inhibits neuronal release of excessive excitatory amino acids [11], reduces calcium overload injury [14, 22], reduces stress induced by reactive nitrogen and oxygen stress [60, 77–79], and inhibits the inflammatory response and apoptosis [7, 78, 80, 81]. Furthermore, BHD acts on glia and neurons to promote release of a variety of cellular nutritional factors to promote neovascularization, nerve regeneration, and synapse formation in the late stage of healing [5, 8–10, 13, 43, 82, 83]. Wang et al. reported [5] that when drugs were given two hours after induction of ischemia, protective effects of BHD were greater than recombinant tissue-type plasminogen activator (r-tPA) administration. Furthermore, genome-wide transcriptome analysis reveals that the neuroprotective effect of BHD on CI/R-induced brain injury in mice may depend on modulation of multiple molecular targets or genes. Therefore, multitargeted neuroprotective actions of BHD may explain our meta-analysis results. Whether it is administered before ischemia or 20 days after ischemia, BHD can improve neurological symptoms in animals. 

The BHD dosage administered ranged from 1.0 g/kg to 60 g/kg, and neuroprotective effects were demonstrated at all dosage levels. Although meta-analysis demonstrated that the greatest effect was exerted in the range of ~25 g/kg, there was no statistically significant difference with the other dose groups. This is consistent with our TCM theory that BHD has a wide effective dose range. A study on pharmacodynamics and acute toxicity showed that oral administration of BHD at a dose of 48 g/kg produced no toxicity in animal experiments [84]. The probability of BHD possessing high toxic or lethal doses is, therefore, unlikely. However, because of the complexity of TCM and BHD ingredients, there may be differences between the active ingredients in the different experimental drug formulations, which creates difficulties in analyzing the precise dose-response relationship. It is therefore necessary to design a larger and more rigorous study, with a standardized BHD formula specifically for this purpose.

No efficacy difference between intraperitoneal and oral administrations of BHD was observed. None of the included studies utilized intravenous administration, so we were unable to compare the efficacy differences between oral and intravenous administrations. Animal species, method of ischemia induction, duration of occlusion, and time to outcome measurement all influence the final effect size of the research, which is consistent with the results of previous studies. Some previous studies [17, 18] suggest that the quality of the research design is an important factor affecting the outcome; however, in our meta-analysis research, there was no significant difference of the effect sizes among the studies with different research design qualities.

### 4.2. Potential Weaknesses of This Analysis

When compared to other candidate chemical drugs in experimental stroke treatment, which have undergone systematic review and meta-analyses, the efficacy of BHD was similar. For example, interleukin-1 receptor antagonist (IL-1RA) reduced infarct volume by 38% [85, 86], erythropoietin by 32% [86], NXY-059 by 43% [87], and G-CSF by 42% [88]. However, in the meta-analysis of this paper, none of the studies investigated stroke in models with comorbidities such as diabetes or hypertension that could exaggerate effect size. This lack of information should certainly be addressed in future studies. Although our choice of stratification variables was prespecified and a stringent significance level was established, some aforementioned results must be interpreted with caution as they may have been due to chance. This meta-analysis possesses other weaknesses. First, meta-analysis can only include available data, and publication bias may result in our analyses overestimating the efficacy of BHD. Furthermore, although we consider that our search strategy is likely to have ascertained most of the relevant publications, it has yet to be validated. 

## 5. Conclusion

While the overall estimate of efficacy from this meta-analysis suggests that BHD possesses substantial neuroprotective action in models of focal cerebral ischemia, for all doses and at each time point of treatment, large studies of high methodological quality (including randomization to treatment group, masked induction of ischemia, and assessment of outcome) are required to produce a precise, unbiased assessment of the efficacy of BHD. The multitarget therapeutic strategy is an important direction for future cerebral ischemia protection treatment, and it requires further in-depth study of TCM compound preparation.

## Figures and Tables

**Table 1:** Design characteristics of included studies.

Study	Species	Stroke model	Method of administration	Outcome measure (treated/control)	Quality score
Bai and Cai [20] 2007	Male, SD rats	Permanent MCAO	14 d after occlusion; i.g.; 11.14 g/Kg, daily	Neurobehavioral score (10/10)	3
Bai et al. [21] 2011	Male, SD rats	Permanent MCAO	14 d after occlusion; i.g.; 11.14 g/Kg, daily	Neurobehavioral score (10/10)	3
Cai et al. [6] 2007	Male, Wistar rats	Temporary MCAO	2 h after occlusion; i.g.; 5 g/Kg, daily	Combined (9/9)	5
Cao and Jiang [22] 2004	Male, SD rats	Temporary MCAO	3 d before occlusion; i.p.; 4 g/Kg, daily	Neurobehavioral score (6/6)	2
Chen et al. [23] 2011	Male, SD rats	Temporary MCAO	7 d before occlusion; i.g.; 40 g/Kg, daily	Neurobehavioral score (18/18)	3
Chu et al. [24] 2005	Male, SD rats	Temporary MCAO	7 d before occlusion; i.g.; 13.3 g/Kg, daily	Neurobehavioral score (11/12)	4
Chu et al. [25] 2006	Male, SD rats	Temporary MCAO	7 d before occlusion; i.g.; 13.3 g/Kg, daily	Combined (11/12)	5
Chu et al. [26] 2006	Male, SD rats	Temporary MCAO	7 d before occlusion; i.g.; 26 g/Kg, daily 13 g/Kg, daily	Neurobehavioral score (20/10)	3
Chu et al. [27] 2011	Male, SD rats	Temporary MCAO	24 h after occlusion; i.g.; 13 g/Kg, daily	Neurobehavioral score (12/12)	4
Chu et al. [8] 2011	Male, KM mice	Temporary MCAO	24 h after occlusion; i.g.; 20 g/Kg, daily	Neurobehavioral score (13/12)	4
Du and Wang [28] 2008	Male, SD rats	? MCAO	Immediately after occlusion; i.g.; 26.6 g/Kg, daily	Neurobehavioral score (10/10)	3
Du et al. [29] 2011	Male, SD rats	Temporary MCAO	2 h after occlusion; i.g.; 14.2 g/Kg, daily	Neurobehavioral score (8/8)	4
Gao and Cai [30] 2004	Male, SD rats	Temporary MCAO	Immediately after occlusion; i.p.; 16 g/Kg, daily	Combined (8/8)	3
Gao et al. [31] 2008	Male, SD rats	Temporary MCAO	5 d before occlusion; i.g.; 13.3 g/Kg, daily	Neurobehavioral score (6/6)	2
Gao et al. [32] 2009	Male, SD rats	Temporary MCAO	5 d before occlusion; i.g.; 13 g/Kg, daily	Neurobehavioral score (6/6)	2
Gao et al. [9] 2009	Male, SD rats	Temporary MCAO	5 d before occlusion; i.g.; 26 g/Kg, daily, 13 g/Kg, daily, and 6.5 g/Kg, daily	Neurobehavioral score (18/6)	3
Guo et al. [33] 2004	Male, SD rats	Temporary MCAO	1 h after occlusion; i.p.; 10 g/Kg, daily	Combined (20/20)	3
Han et al. [34] 2001	Male, Wistar rats	Photothrombotic MCAO	7 d before occlusion; i.g., 12.8 g/Kg, daily	Infarction size (8/8)	3
Jiang and Zhang [35] 2004	Male, SD rats	Temporary MCAO	2 h after occlusion; i.p.; 4.9 g/Kg, daily	Infarction size (10/10)	4
Jiang et al. [36] 2005	Male, SD rats	Temporary MCAO	Immediately after occlusion; i.p.; 5.8 g/Kg, daily	Neurobehavioral score (6/6)	4
Jiang et al. [14] 2005	Male, SD rats	? MCAO	5 d before occlusion; i.p.; 29 g/Kg, daily	Neurobehavioral score (6/6)	3
Li and Cai [13] 2007	Male, SD rats	Temporary MCAO	2 h after occlusion; i.g.; 14 g/Kg, daily	Combined (5/5)	3
Li and Wang [37] 2011	Male, SD rats	Temporary MCAO	4 d after occlusion; i.g.; 6.4 g/Kg, daily	Neurobehavioral score (10/10)	4
Liang and Zhang [38] 2010	Male, SD rats	? MCAO	24 h after occlusion; i.g.; 7.15 g/Kg, daily	Combined (15/15)	2
Liao and Tong [15] 2004	Male, SD rats	Temporary MCAO	7 d before occlusion; i.g.; 12.96 g/Kg, daily	Combined (6/6)	3
Liu and Wu [39] 2004	Male, SD rats	Temporary MCAO	7 d before occlusion; i.g.; 20 g/Kg, daily and 10 g/Kg, daily	Combined (20/10)	5
Liu et al. [40] 2005	Male, SD rats	Temporary MCAO	7 d before occlusion; i.g., 20 g/Kg, daily and 10 g/Kg, daily	Infarction size (20/10)	3
Liu and Peng [41] 2005	Male, Wistar rats	Permanent MCA cauterization	20 d after occlusion; i.g.; 14 g/Kg, daily	Neurobehavioral score (8/8)	3
Liu et al. [42] 2007	Male, Wistar rats	Permanent MCAO	2 h after occlusion; i.g.; 14.2 g/Kg, daily	Neurobehavioral score (8/8)	4
Liu et al. [43] 2008	Mixed, Wistar rats	Permanent MCAO	2 h after occlusion; i.g.; 13.8 g/Kg, daily	Neurobehavioral score (5/5)	3
Liu et al. [44] 2012	Mixed, SD rats	Permanent MCAO	4 d before occlusion; i.g.; 13 g/Kg, daily	Combined (8/8)	4
Lu and Peng [45] 2008	Male, Wistar rats	Permanent MCA cauterization	15 d after occlusion; i.g.; 50 g/Kg, daily, 25 g/Kg, daily, and 12.5 g/Kg, daily	Neurobehavioral score (27/9)	3
Ma and Fang [46] 2009	Male, SD rats	Temporary MCAO	7 d before occlusion; i.g.; 60 g/Kg, daily	Combined (10/10)	4
Mo and Zheng [47] 1997	Mixed, S rats	Permanent MCA cauterization	7 d before occlusion; i.g.; 13.8 g/Kg, daily	Infarction size (7/6)	3
Su et al. [48] 2012	Male, SD rats	Temporary MCAO	24 h after occlusion; i.g.; 12 g/Kg, daily	Combined (18/18)	3
Sun and Peng [49] 2004	Male, Wistar rats	Permanent MCA cauterization	20 d after occlusion; i.g.; 50 g/Kg, daily, 25 g/Kg, daily, and 12.5 g/Kg, daily	Neurobehavioral score (27/9)	4
Sun et al. [50] 2010	Male, SD rats	Temporary MCAO	7 d before occlusion; i.g.; 25.7 g/Kg, daily	Neurobehavioral score (16/16)	3
Tan et al. [51] 2006	Male, Wistar rats	Permanent MCA cauterization	20 d after occlusion; i.g.; 25.66 g/Kg, daily and 6.42 g/Kg, daily	Neurobehavioral score (16/8)	3
Tian and Liu [52] 2010	Male, SD rats	? MCAO	2 h after occlusion; i.g.; 6 g/Kg, daily	Combined (5/5)	4
Wang et al. [53] 2005	Male, SD rats	Temporary MCAO	5 d before occlusion; i.g.; 20 g/Kg, daily and 10 g/Kg, daily	Combined (28/14)	4
Wang et al. [54] 2006	Male, SD rats	Temporary MCAO	7 d before occlusion; i.g.; 24 g/Kg, daily	Combined (10/10)	3
Wang et al. [55] 2010	Male, Wistar rats	Embolic MCAO	24 h after occlusion; i.g.; 10 g/Kg, daily	Neurobehavioral score (8/8)	2
Wang et al. [5] 2011	Male, ICR mice	Temporary MCAO	2 h after occlusion; i.g.; 2 g/Kg, daily and 1 g/Kg, daily	Infarction size (40/20)	6
Wei et al. [56] 2010	Male, Wistar rats	Temporary MCAO	3 d before occlusion; i.g.; 10 g/Kg, daily	Combined (8/8)	5
Wu and Luo [57] 2008	Male, SD rats	Temporary MCAO	1 h after occlusion; i.g.; 13.3 g/Kg, daily	Combined (16/16)	3
Xu and Liao [58] 2006	Male, Wistar rats	Temporary MCAO	Immediately after occlusion; i.p.; 10 g/Kg, daily	Combined (8/8)	4
Xue [59] 2006	Male, SD rats	Temporary MCAO	3 d before occlusion; i.g.; 8.4 g/Kg, daily and 4.2 g/Kg, daily	Infarction size (20/10)	3
Yang [60] 2010	Male, SD rats	Temporary MCAO	7 d before occlusion; i.g.; 13 g/Kg, daily, 4.3 g/Kg, daily, and 1.45 g/Kg, daily	Combined (30/10)	3
Yi et al. [61] 2010	Male, Wistar rats	? MCAO	2 h after occlusion; i.g.; 14.2 g/Kg, daily	Combined (10/10)	4
Yin and Cai [16] 2007	Male, SD rats	Permanent MCAO	2 h after occlusion; i.p.; 5.9 g/Kg, daily	Neurobehavioral score (10/10)	2
Yin and Wu [62] 2012	Male, gerbils	Temporary MCAO	7 d before occlusion; i.g.; 51 g/Kg, daily	Infarction size (5/5)	3
Zhang et al. [63] 2009	Male, Wistar rats	? MCAO	7 d before occlusion; i.g.; 25 g/Kg, daily	Combined (10/10)	2
Zhao et al. [64] 2005	Male, SD rats	Temporary MCAO	0.5 h after occlusion; i.g.; 1.4 g/Kg, daily	Combined (8/8)	3
Zhao and Qu [65] 2007	Male, SD rats	Temporary MCAO	7 d before occlusion; i.g.; 12.8 g/Kg, daily	Infarction size (6/6)	2
Zhao et al. [11] 2012	Mixed, Wistar rats	Temporary MCAO	0.5 h, 1 h, 2 h, 4 h, and 6 h after occlusion; i.g.; 40 g/Kg, daily	Infarction size (35/7)	6
Zhou and Cai [66] 2012	Male, SD rats	Permanent MCAO	2 h after occlusion; i.g.; 5 g/Kg, daily	Combined (15/15)	3

Note: (a) stroke model: temporary or permanent MCAO (suture-occluded method), permanent MCA cauterization; photothrombotic MCA; embolic MCA.

(b) Method of administration: time of administration; route of drug delivery; dose range given in the first 24 h.

i.g.: intragastric administration; i.p.: intraperitoneal injection. MCAO: middle cerebral artery occlusion; MCA: middle cerebral artery.

**Table 2:** Possible protective mechanisms of BHD.

Possible drug protective mechanism	Studies
Hemorheology and cerebral circulation improvement	[5, 34, 47]
Cerebral edema relief; blood brain barrier permeability reduction	[28, 30, 39, 40, 44, 45, 53, 60, 61]
Excitatory neurotransmitter toxicity reduction	[11]
Reduction of Ca^2+^ overload	[14, 22]
Oxidative stress and nitration stress reaction reduction	[15, 16, 38, 60]
Antiinflammatory effect	[5, 26, 31, 46, 63]
Antiapoptotic effect	[5, 15, 38, 40, 50]
Promotion of new blood vessel formation	[5, 6, 8, 27, 37, 42, 45, 52, 62]
Promotion of nerve regeneration and synapse formation	[5, 9, 13, 16, 20, 29, 32, 41, 43, 48, 51, 55, 57, 59, 66]
Other	[22–25, 33, 36, 49, 64]

## References

[1] O’Collins VE, Macleod MR, Donnan GA, Horky LL, Van Der Worp BH, Howells DW (2006). 1,026 Experimental treatments in acute stroke. *Annals of Neurology*.

[2] Savitz SI, Fisher M (2007). Future of neuroprotection for acute stroke: in the aftermath of the SAINT trials. *Annals of Neurology*.

[3] Wang QR (2005). *Yilin Gaicuo*.

[4] Johnston L Buyang Huanwu Decoction. http://www.healingtherapies.info/Buyang.htm.

[5] Wang HW, Liou KT, Wang YH (2011). Deciphering the neuroprotective mechanisms of Bu-yang Huan-wu Decoction by an integrative neurofunctional and genomic approach in ischemic stroke mice. *Journal of Ethnopharmacology*.

[6] Cai G, Liu B, Liu W (2007). Buyang Huanwu Decoction can improve recovery of neurological function, reduce infarction volume, stimulate neural proliferation and modulate VEGF and Flk1 expressions in transient focal cerebral ischaemic rat brains. *Journal of Ethnopharmacology*.

[7] Li XM, Bai XC, Qin LN, Huang H, Xiao ZJ, Gao TM (2003). Neuroprotective effects of Buyang Huanwu Decoction on neuronal injury in hippocampus after transient forebrain ischemia in rats. *Neuroscience Letters*.

[8] Chu LS, Yin YJ, Ke Q, Chen WY, Chen FM (2011). Effect of buyanghuanwu Decoction on angiogenesis and Ang-1/Tie-2 expression after focal cerebral ischemia in mice. *Chinese Journal of Behavioral Medicine and Brain Science*.

[9] Gao J, Lü F, Zhu C (2009). Effects of Buyang Huanwu Decoction on cell proliferation and differentiation in the hippocampal dentate gyrus of aged rats following cerebral ischemia/reperfusion. *Neural Regeneration Research*.

[10] Cai G, Liu B (2010). Buyang Huanwu Decoction increases vascular endothelial growth factor expression and promotes angiogenesis in a rat model of local cerebral ischemia. *Neural Regeneration Research*.

[11] Zhao LD, Wang JH, Jin GR, Zhao Y, Zhang HJ (2012). Neuroprotective effect of Buyang Huanwu Decoction against focal cerebral ischemia/reperfusion injury in rats—time window and mechanism. *Journal of Ethnopharmacology*.

[12] Hao CZ, Wu F, Shen JG (2012). Clinical efficacy and safety of Buyang Huanwu Decoction for acute ischemic stroke: a systematic review and meta-analysis of 19 randomized controlled trials. *Evidence-Based Complementary and Alternative Medicine*.

[13] Li X, Cai GX (2007). *Study on the mechanism of synapse-1 remodeling in the cerebral ischemia rats with Buyang Huanwu Decoction [M.S. thesis]*.

[14] Jiang HY, Shan LL, Cao P, Guo SJ (2005). Neuroprotection of Qileng Decoction on focal cerebral ischemia-reperfusion injury in rats. *Journal of Emergency in Traditional Chinese Medicine*.

[15] Liao CL, Tong L (2004). *Mechanism studies of protective effect of BuyangHuanwu Decoction on experimental cerebral ischemic injury [M.S. thesis]*.

[16] Yin TL, Cai GX (2007). *Protective effects and mechanisms of Caowei-Buyang Huanwu Decoction against neuronal Injury in MCAO rat [Ph.D. thesis]*.

[17] Macleod MR, O’Collins T, Horky LL, Howells DW, Donnan GA (2005). Systematic review and metaanalysis of the efficacy of FK506 in experimental stroke. *Journal of Cerebral Blood Flow and Metabolism*.

[18] Macleod MR, O’Collins T, Howells DW, Donnan GA (2004). Pooling of animal experimental data reveals influence of study design and publication bias. *Stroke*.

[19] Horn J, De Haan RJ, Vermeulen M, Luiten PGM, Limburg M (2001). Nimodipine in animal model experiments of focal cerebral ischemia: a systematic review. *Stroke*.

[20] Bai XS, Cai GX (2007). *Effect of micropowder Buyang Huanwu Decoction on neurologic deficits and Nogo-a of MCAO model rat [Ph.D. thesis]*.

[21] Bai XS, Cai GX, Yin TL (2011). A pharmacology method to estimate effect of Buyang Huanwu Decoction on neurologic deficits of MCAo Model Rats. *World Chinese Medicine*.

[22] Cao P, Jiang HY (2004). Effect of daqinjiao Decoction, zhenganxifeng Decoction and Buyang Huanwu Decoction on cerebral ischemia and reperfusion injuries in rats. *Journal of Traditional Chinese Medicine University of Hunan*.

[23] Chen W, Wang H, Zhang SW Effect of trichosanthin on high plasma homocystein in rats with cerebral ischemia-reperfusion. *Journal of Emergency in Traditional Chinese Medicine*.

[24] Chu LS, Yang WM, Shao L, Meng DY (2005). Effect of Buyanghuanwu Decoction on learning and memory in rats following focal cerebaral ischemia injury. *Chinese Journal of Behavioral Medical Science*.

[25] Chu LS, Shao L, Meng DY, Li JH, Sun J (2006). Long-term cerebroprotective effects of Buyang Huanwu Decoction in rats after focal cerebral ischemia injury. *Chinese Journal of Clinical Rehabilitation*.

[26] Chu LS, Li JH, Sun J, Yang WM, Ke Q (2006). Effects of Buyanghuanwu Decoction on infiltration of neutrophils and expression of ICAM-1 after middle cerebral artery occlusion in rats. *Journal of Zhejiang University of Traditional Chinese Medicine*.

[27] Chu LS, Jiang YY, Ke Q, Yin YJ, Chen WY (2011). Buyanghuanwu Decoction enhances angiogenesis and improves functional recovery after focal cerebral ischemia in rats. *Chines Archives of Traditional Chinese Medicine*.

[28] Du ZG, Wang YL (2008). Effects of Buyanghuanwu tang formula on AQP-4 of rats with experimental ischemic brain. *World Science and Technology*.

[29] Du K, Liu BY, Zeng R, Yi J, Cai GX (2011). Effect of ultramicro-Naodejian on GAP-43 and cerebral nerve regeneration after focal cerebral ischemia in rats. *Hunan Journal of Traditional Chinese Medicine*.

[30] Gao Y, Cai Df (2004). *Effect of BuYangHuanWu recipe on the cerebral microcirculation in MCAO rat after reperfusion [Ph.D. thesis]*.

[31] Gao Jf, Wang XH, Liu K, Lu M (2008). Influence of Buyanghuanwu Decoction on expression changes of cerebral TNF-a, ICAM-1 and VCAM-1 in aged rats with cerebral ischemia-reperfusion lesion. *Journal of Beijing University of Traditional Chinese Medicine*.

[32] Gao JF, Lu FH, Wang XH (2009). Influence of Buyang Huanwu Decoction on dentate gyrus cell proliferation and differentiation in senile rats with cerebral ischemia-reperfusion lesion. *Chinese Journal of Gerontology*.

[33] Guo NY, Wei W, HU LJ (2004). The effects of Injection of Buyanghuanwutang on volume of infarction and changes of electroencephalography. *Journal of Clinical Internal Medicine*.

[34] Han D, Liao FL, Li W, Liang RX, Ying XJ (2001). Effect of BuYanHanWu (BYHW) Tang upon infarction, vessel damage penumbra (VDP) fibrinolytic system activity and endothelin. *China Journal of Experimental Traditional Medical Formulae*.

[35] Jiang HY, Zhang SW (2004). Pathomorphology study of qileng Decoction on focal cerebral ischemia and reperfusion in rats. *China Journal of Traditional Chinese Medicine and Pharmacy*.

[36] Jiang HY, Yu QH, Cao P, Deng SF (2005). Effects of Qileng Decoction on glial fibrillary acidic protein expression in the ischemic penumbra of rats following cerebral ischemia/reperfusion. *Chinese Journal of Integrated Traditional and Western Medicine in Intensive and Critical Care*.

[37] Li J, Wang CY (2011). Experimental study of Buyang Huanwu Decoction on angiogenesis after cerebaral ischemia reperfusion injury in rats. *Journal of Emergency in Traditional Chinese Medicine*.

[38] Liang YK, Zhang JR (2010). *Study on the method of Nourishing yin, filling Pith and benifiting brain towards the Protective mechanism of rat focal cerebral ischemia-reperfusion chronic injury [M.S. thesis]*.

[39] Liu C, Wu JL (2004). Protection of Buyang Huanwu Decoction on the change of behavior and pathology of focal cerebral ischemia re-perfusion injury in rats. *Hubei Journal of Traditional Chinese Medicine*.

[40] Liu C, Wu JL, Chang EZ, Cai F (2005). Effect of Buyang Huanwu Decoction on apoptosis after focal cerebral ischemia-reperfusion in rats. *Chinese Journal of Gerontology*.

[41] Liu NP, Peng K (2005). *Effect of Buyang Huanwu Decoction on the change of motor function and structure of neurons synapse in brain tissue of the stroke sequela models of rats which are lack of Qi and with blood silted [M.S. thesis]*.

[42] Liu BY, Cai GX, Liu W, Chen XM (2007). Effect of Buyang Huanwu Decoction on vascular endothelial growth factor and its receptor Flk1 in rats after focal cerebral ischemia. *Chinese Traditional and Herbal Drugs*.

[43] Liu F, Bai XS, Liu BY, Cai GX (2008). Effect of Bu Yang Huan wu Decoction on basic fibroblast growth factor in rats with cerebral ischemia. *Chinese Journal of Integrated Traditional and Western Medicine in Intensive and Critical Care*.

[44] Liu JX, Li JS, Yu W, Hei CC, Liu HX, Ren FF (2012). Effects of Xinglou Chengqi Decoction and Buyang Huanwu Decoction on the injury of hippocampal neurons in rats with cerebral ischemia. *Chinese Journal of Experimental Traditional Medical Formulae*.

[45] Lu YK, Peng K (2008). *Study of expression of vascular endothelial growth factor and effect of BuYang HuanWu decotion on the stroke sequela models of rats with deficiencyof Qi and blood stasis [M.S. thesis]*.

[46] Ma TG, Fang JK (2009). *Experimental study of different quantity ratio of Huang Qi in Buyanghuanwu Decoction on cerebral infarciton [degree paper]*.

[47] Mo SL, Zheng YS (1997). Effect of Buyang Huanwu disassembled prescriptions on brain cortical infarct size and plasma endothelin level in experimental cerebral ischemic injury rats. *Pharmacology and Clinics of Chinese Materia Medica*.

[48] Su XH, Kong XY, Pang ZR, Lin N (2012). Effect of Buyang Huanwu Decoction on migration of neural stem cells after focal cerebral ischemia in rats. *Chinese Journal of Experimental Traditional Medical Formulae*.

[49] Sun Z, Peng K (2004). Effect of buyang huanwu tang on Na^+^, K^+^, Ca^2+^ and Mg^2+^ contents in the brains of rats with deficiency of qi and blood stasis after stroke. *Chinese Journal of Clinical Rehabilitation*.

[50] Sun LQ, Zhao YN, Li JM, Cui JZ, Zhang YX (2010). Buyanghuanwu recipe inhibits neuronal apoptosis after cerebral ischemia/reperfusion injury possibly through the p38MAPK/COX2 signal pathway in rats. *China Journal of Modern Medicine*.

[51] Tan XH, Qu HD, Peng K (2006). Effects of Buyanghuanwu Decoction on nerve proliferation in rats with sequelae of ischemic stroke. *Journal of Southern Medical University*.

[52] Tian ZH, Liu BY (2010). Effect of buyangHuanwu Decoction on angiogenesis in rats after focal cerebral ischemia. *Chinese Journal of Arteriosclerosis*.

[53] Wang XG, Tong ET, Sun SG (2005). Protection effects of buyang huanwu decocton on cerebral ischemia and reperfusion injures. *Zhejiang Journal of Integrated Traditional Chinese and Western Medicine*.

[54] Wang Y, Wang YL, Feng X, Chen CL (2006). A Comparative study on the treatment of experimental ischemic stroke with Buyang Huanwu Decoction or daqinjiao Decoction. *Shanxi Journal of Traditional Chinese Medicine*.

[55] Wang HT, Yang MF, Cao XL, Sun BL (2010). Effect of Buyang Huanwu Decoction combined with sports training on neurons synapse remodeling in rats with focal cerebral infarction. *Chinese Journal of Experimental Traditional Medical Formulae*.

[56] Wei SC, Li WQ, Wang ZW, Gao JD (2010). Influence of ultrafiltration to protective effect of buyang-huanwu Decoction on the cerebral ischemia-reperfusion injury. *Lishizhen Medicin and Materia Medica Research*.

[57] Wu HY, Luo R (2008). *The effects of pulsed magnetic field and Buyanghuanwu Decoction on IGF-1 expression in cerebral ischemia reperIusion rats [Ph.D. thesis]*.

[58] Xu YL, Liao L (2006). Effects of method of supplement qi and activating blood cirulation and channel combined with mild-hypothermia on neurlogic deficits and infarct size in cerebral infarction model rats of qi-deficiency and blood. *Traditional Chinese Medicine Journal*.

[59] Xue YH (2006). Effect of buyanghuanwu liquid on the content of Insulin-like growth factor-I (IGF-I) and brain-derived neurotrophic factor (BDNF) in focal cerebral ischemia and reperfusion rats. *Chinese Journal of the Practical Chinese With Modern Medicine*.

[60] Yang CG (2010). Protective effect of buyang-huanwu Decoction on the cerebral ischemia-reperfusion injury in rats. *Research and Practice on Chinese Medicine*.

[61] Yi J, Huan X, Xie Y, Yang Y, Chen XM, Liu BY (2010). Effect of naodejian Decoction on nerve function and infarct volume in rats after focal cerebral ischemia. *Journal of TCM University of Hunan*.

[62] Yin YH, Wu XG (2012). Study on effect of Buyang Huanwu Decoction on expression of VEGF and Ang-1 in gerbils with cerebral ischemia-reperfusion. *Journal of Clinical and Experimental Medicine*.

[63] Zhang L, Li R, Chen J, Ma TG (2009). Effect of Buyang Huanwu Decoction on cerebral infarction volume, nerve function and Serum IL-10 level in focal cerebral ischemia rats. *China Journal of Traditional Chinese Medicine and Pharmacy*.

[64] Zhao H, Zhang QJ, Wang T, Xu X, Liu Y (2005). Effect of zhongfen jiedu Decoction on cerebral infarction area and nerve deficit in acute cerebral ischemia rats. *Shanghai Journal of Traditional Chinese Medicine*.

[65] Zhao YL, Qu YZ (2007). Influence of Buyang Huanwu Decoction on cerebral infarct volume and pathological changes after cerebral ischemia and reperfusion. *Chinese Journal of Information on Traditional Chinese Medicine*.

[66] Zhou SN, Cai GX (2012). *The effects of Buyang Huanwu Decoction on proliferation of nerve cell and expressions of TLR2, TLR4, MyD88, NF-KBp65 in rats with cerebral ischemia [Ph.D. thesis]*.

[67] Deng CQ, Xiao SH, Ge JW, Ji AG (1992). Effects of Buyang Huanwu Decoction on injury of cerebral ischemic reperfusion in gerbils. *Journal of Hunan College of Traditional Chinese Medicine*.

[68] Ginsberg MD (2009). Current status of neuroprotection for cerebral ischemia synoptic overview. *Stroke*.

[69] Moskowitz MA, Lo EH, Iadecola C (2010). The science of stroke: mechanisms in search of treatments. *Neuron*.

[70] Barone FC (2009). Ischemic stroke intervention requires mixed cellular protection of the penumbra. *Current Opinion in Investigational Drugs*.

[71] Faraci FM (2006). Reactive oxygen species: influence on cerebral vascular tone. *Journal of Applied Physiology*.

[72] Feuerstein GZ, Wang X (2001). Inflammation and stroke: benefits without harm?. *Archives of Neurology*.

[73] Ceulemans AG, Zgavc T, Kooijman R, Hachimi-Idrissi S, Sarre S, Michotte Y (2010). The dual role of the neuroinflammatory response after ischemic stroke: modulatory effects of hypothermia. *Journal of Neuroinflammation*.

[74] Hoyte L, Barber PA, Buchan AM, Hill M (2004). The rise and fall of NMDA antagonists for ischemic stroke. *Current Molecular Medicine*.

[75] Savitz SI, Schäbitz WR (2008). A critique of SAINT II: wishful thinking, dashed hopes, and the future of neuroprotection for acute stroke. *Stroke*.

[76] Lo EH (2008). Experimental models, neurovascular mechanisms and translational issues in stroke research. *British Journal of Pharmacology*.

[77] Guo P, Wang J, Wang H (2002). Study of the effects of Bu Yang Huan Wu Tang on SOD and MDA brain tissue in cerebral ischemia and reperfusion of rats. *Chinese Journal of Applied Physiology*.

[78] Lin R, Liu Y, Shi X (2011). The roles of Buyang Huanwu Decoction in anti-inflammation, antioxidation and regulation of lipid metabolism in rats with myocardial ischemia. *Evidence-Based Complementary and Alternative Medicine*.

[79] Wang L, Jiang DM (2009). Neuroprotective effect of Buyang Huanwu Decoction on spinal ischemia/reperfusion injury in rats. *Journal of Ethnopharmacology*.

[80] Fan L, Wang K, Cheng B (2006). Effects of Buyang Huanwu Decoction on apoptosis of nervous cells and expressions of Bcl-2 and Bax in the spinal cord of ischemia-reperfusion injury in rabbits. *Journal of Traditional Chinese Medicine*.

[81] Yang G, Fang Z, Liu Y (2011). Protective effects of Chinese traditional medicine Buyang Huanwu Decoction on myocardial injury. *Evidence-Based Complementary and Alternative Medicine*.

[82] Sun J, Bi Y, Guo L (2007). Buyang Huanwu Decoction promotes growth and differentiation of neural progenitor cells: using a serum pharmacological method. *Journal of Ethnopharmacology*.

[83] Zhang YK, Han XY, Che ZY (2010). Effects of Buyang Huanwu Tang combined with bone marrow mesenchymal stem cell transplantation on the expression of VEGF and Ki-67 in the brain tissue of the cerebral ischemia-reperfusion model rat. *Journal of Traditional Chinese Medicine*.

[84] Chen SQ, Yan F, Xu DJ, Liu XY, Chen SJ (2000). Studies on pharmacodynamics and acute toxicity of buyang huanwu tang granule. *West China Journal of Pharmaceutical Sciences*.

[85] Banwell V, Sena ES, Macleod MR (2009). Systematic Review and Stratified Meta-analysis of the Efficacy of Interleukin-1 Receptor Antagonist in Animal Models of Stroke. *Journal of Stroke and Cerebrovascular Diseases*.

[86] Minnerup J, Heidrich J, Rogalewski A, Schäbitz WR, Wellmann J (2009). The efficacy of erythropoietin and its analogues in animal stroke models: a meta-analysis. *Stroke*.

[87] MacLeod MR, Van Der Worp HB, Sena ES, Howells DW, Dirnagl U, Donnan GA (2008). Evidence for the efficacy of NXY-059 in experimental focal cerebral ischaemia is confounded by study quality. *Stroke*.

[88] Minnerup J, Heidrich J, Wellmann J, Rogalewski A, Schneider A, Schabitz WR (2008). Meta-analysis of the efficacy of granulocyte-colony stimulating factor in animal models of focal cerebral ischemia. *Stroke*.

